# Supplementary material for: Non-Invasive Mapping of Cerebral Autoregulation Using Near-Infrared Spectroscopy: A Study Protocol
Source: Methods Protoc. 2023 Jun 9;6(3):58. doi: 10.3390/mps6030058 (PMC10304987; doi:10.3390/mps6030058)
Supplement: Supplementary file 1 [file mps-06-00058-s001.zip › supplementary File S2.pdf]

Rady Faculty of Health Sciences | University of Manitoba

Max Rady College of Medicine
Department of Surgery
Section of Neurosurgery
Health Sciences Centre
GB1 – 820 Sherbrook Street
Winnipeg, Manitoba R3A 1R9
Canada
Fax: 204-783-7356

Volunteers Needed!

Interested in Participating in Neuroscience Research? Interested in Having Your Brain Monitored Using Cutting-Edge Technology?

We are actively looking for participants aged 18 and older to participate in the healthy volunteer study entitled:
*“**Non-Invasive Mapping of Cerebral Autoregulation Using Near Infrared Spectroscopy: A Healthy Control Study**”*

This study is run out of Dr. Frederick Zeiler’s cerebrovascular physiology laboratory at the University of Manitoba. It involves 60 minutes of your time to have various aspects of brain physiology continuously monitored non-invasively through static recording and perturbation testing (orthostatic challenge, vascular chemo-reactivity, and neurovascular coupling impact). Various types of non-invasive monitors will be applied simultaneously, including: non-invasive arterial blood pressure, Near Infrared Spectroscopy, and Respiratory Monitor.

To be eligible you need to:

1. Be 18 years or older
2. Have no history of neurological conditions
3. Have no history of cardiovascular conditions (i.e., stroke, heart disease, peripheral vascular disease)

Volunteers will receive reimbursement for their participation.

For more information email: amanjyot.sainbhi@umanitoba.ca
call: 1-204-787-2909
(To reach Dr. Zeiler’s Laboratory Team)
