# Supplementary material for: Non-Invasive Mapping of Cerebral Autoregulation Using Near-Infrared Spectroscopy: A Study Protocol
Source: Methods Protoc. 2023 Jun 9;6(3):58. doi: 10.3390/mps6030058 (PMC10304987; doi:10.3390/mps6030058)
Supplement: Supplementary file 1 [file mps-06-00058-s001.zip › supplementary File S3.pdf]

Rady Faculty of Health Sciences

Department of Surgery
Section of Neurosurgery
Health Sciences Centre
GB127C – 820 Sherbrook Street
Winnipeg, MB R3A 1R9
T: (204) 787-7261
F: (204) 787-3851

# RESEARCH PARTICIPANT INFORMATION AND CONSENT FORM – Healthy Volunteer Objective 1

**Title of Study:**
Non-Invasive Mapping of Cerebral Autoregulation Using Near Infrared Spectroscopy

**Principal Investigator:**
Dr. Frederick A. Zeiler, BSc MD PhD CIP FRCSC
Associate Professor, Departments of Surgery,
Human Anatomy and Cell Science, Biomedical Engineering
University of Manitoba
Health Sciences Centre
GF231 - 820 Sherbrook Street
Winnipeg, Manitoba
R3A 1R9
Phone: +1 (431) 335-7461

**Sponsor:**
NSERC DG (RGPIN-2022-03621) and Health Sciences Centre Foundation (HSFC) MPI Research Operating Grant

**You are being asked to participate in a research study. Please take your time to review this consent form and discuss any questions you may have with the study staff. You may take your time to make your decision about participating in this study and you may discuss it with your friends, family or (if applicable) your doctor before you make your decision. This consent form may contain words that you do not understand. Please ask the study staff to explain any words or information that you do not clearly understand.**

## Purpose of Study

This research study is being conducted to optimize the device and perform testing on healthy volunteers. A block-trial design will be used to evaluate the cerebral mapping system's performance to map cerebral blood flow during various interventions (i.e. neck artery compressions, orthostatic change, $CO_2$ concentration change and neurovascular assessment).

We will use non-invasive near infrared spectroscopy (NIRS) system with non-invasive continuous arterial blood pressure (niABP) monitor to create a new wearable and portable imaging system. This system will be able to derive blood flow maps of the entire brain with high sampling rates. We hope to be able to demonstrate the effectiveness of non-invasively monitoring the brain's blood vessels in healthy patients and provide a normal value range for these measures. This could significantly change the way patients will be monitored with and without brain injury in both Manitoba and across the world, and potentially improve the ability to predict outcome.

A total of 50 participants will participate in this study.

**Study procedures**

If you take part in this study, you will have the following procedures:

A member of the research team will approach you for consent to be included in the study.

To be eligible for this study you need to be:

1. An adult (age 18 or older)
2. No prior history of neurological condition
3. No prior history of stroke or brain disorder

By consenting to this study you will receive 90 minutes of entirely non-invasive brain and blood pressure monitoring. By non-invasive, this means that there is no procedure required to place the monitor, or needle pokes. The monitors are attached by head cap (shown in Figure 1), a separate medical grade NIRS device (INVOS 5100C/7100) will be attached to the forehead via a non-invasive single sticky pad, finger cuffs, nose clip or placed near the skin surface with ultrasound gel. The monitors will continuously monitor the brain's blood flow, and oxygen delivery.

Figure 1: Multi-channel NIRS system

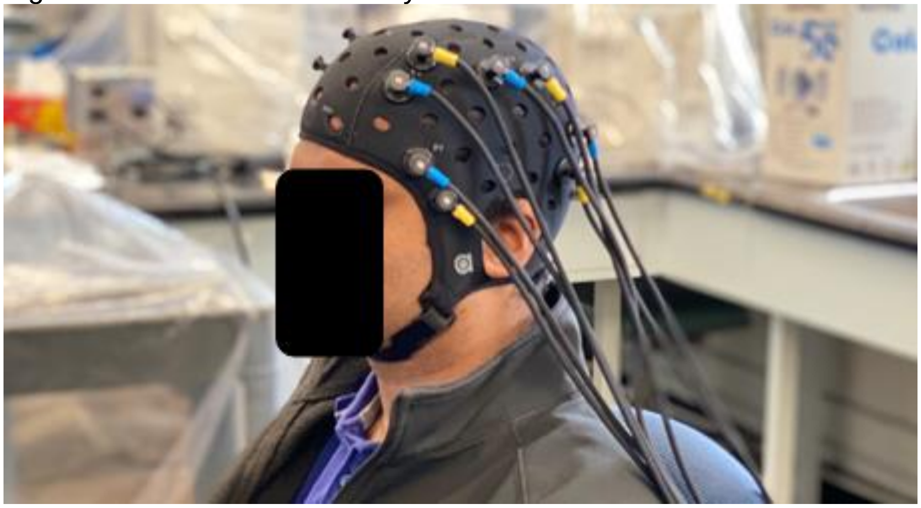

The multi-channel NIRS system (OxyMon Mk III, Artinis Medical Systems, Elst, Netherlands) is shown which provides high-frequency oxy/deoxy-hemoglobin outputs at each channel with customizable layouts.

The block design will consist of four blocks of different interventions with a baseline rest between each block varying from 1-3 minutes depending on the time required to go back to normal levels after intervention. The length of each block will vary depending on the number of repetitions along with the time required to go back to normal levels.

First, the transient hyperemic response will be tested by compressing neck artery to reduce blood flow velocity to 50% accessed using transcranial doppler (TCD) probe. This test will consist of 5 compressions lasting 5 seconds each with a 60 second interval between each compression to allow normalization of the CBF to precompression levels, and the total time for this block would be 6 minutes.

Second, the orthostatic challenge response will be evaluated by lying-to-sit and sit-to-stand methods 5 times where sitting and standing positions will be held for 3 minutes each for a total block time of 35 minutes. A baseline in lying position will be collected at the start of the block, then the position will be changed from lying to sit for 3 minutes and then position will be changed to standing for another 3 minutes.

Third, vascular chemo-reactivity will be assessed by varying $CO_2$ concentrations through slow and fast breathing exercises accessed through nose clip. The breathing trials will occur two times and each of them will have fast and slow trials along with an interval of normal breathing. First time, the normal, fast, and slow breathing trials will be 2.5 minutes long while the second time, these breathing trials will be 1.5 minutes long. This block will take a total time of 12 minutes.

The fourth block will evaluate the neurovascular coupling using Automated Neuropsychological Assessment Metrics (ANAM) General Neuropsychological Screening (GNS) Clinical Toolkit on the computer which contains 5 tests and the volunteer will need to perform tasks such as identifying target letter in a sequence, indicating which hand the man being displayed is holding a ball, tracking a moving circle with a "+" inside where the pointer should remain inside the circle, as close to "+" as possible, solving a mathematical problem, and reading a word or colour of the word. This last Neurovascular coupling block is expected to be completed in 20 minutes.

All data from the devices will be recorded onto laptops for analysis at a later date, Both the information from these brain monitors and blood pressure are used to determine the brain's ability to regulate blood flow.

The researcher may decide to take you off this study if there is sufficient reasonable cause. A written notification documenting the reason for study suspension or termination will be provided to the REB. The circumstances that may warrant termination include but are not limited to insufficient adherence to protocol requirements and data that are not sufficiently complete.

You can stop participating at any time. However, if you decide to stop participating in the study, we encourage you to talk to the study staff first.

*Volunteers who participated in the study will be given a short version of the published results in layman's terms following completion of the study.*

**<u>Risks and Discomforts</u>**

There is no potential harm to participants since the NIRS OxyMon, NIRS INVOS 5100C/7100, and Finapres tools are entirely safe and non-invasive devices. The NIRS INVOS 5100C/7100, and Finapres system are Health Canada approved clinical monitoring devices and functions using a sticky pad sensor to measure oxygen delivery to the brain and a finger-cuff based assessment of blood pressure, respectively. The NIRS system is a research tool and uses entirely non-invasive low-intensity light absorption to measure hemoglobin and oxy-hemoglobin concentrations to a maximum depth of ~4 cm. Such devices have been used to measure oxy-hemoglobin in healthcare settings for ~50 years (i.e. they form the basis for finger-based oxygen saturation and brain oxygen saturation probes used every day around the world). Given this research tool is not a medical device it is not under Health Canada's jurisdiction and is considered a research

instrument for exercise physiology research applications.

Although NIRS and niABP have no side effects but there can be side effects from the interventions (i.e. temporary carotid compression, orthostatic challenge, and hypocapnia/hypercapnia) performed in both objectives. The side effects of these interventions can be feeling lightheaded/nauseated (presyncope) or fainting (syncope). Since the healthy volunteers will be screened beforehand to have no history of neurological illness, systemic vascular or cerebrovascular disease, it reduces the likelihood of presyncope/syncope. During the data collection, the heart rate, blood pressure, and respiratory rate will be recorded and monitored for abnormal values based on the tests. The testing will be halted if heart rate, blood pressure, or respiratory rate goes above or below the expected range, or if the volunteers start to feel uneasy or showing previously mentioned symptoms.

**Benefits**

There will be no direct benefit to you from participating in this study. We hope the information learned from this study will benefit other people in the future.

**Costs**

All the procedures, which will be performed as part of this study, are provided at no cost to you.

**Payment for participation**

All participants will receive a $20.00 CAD honorarium for participation, in the form of a gift card to one of the local coffee shops.

**Confidentiality**

Information gathered in this research study may be published or presented in public forums, however your name and other identifying information will not be used or revealed. Despite efforts to keep your personal information confidential, absolute confidentiality cannot be guaranteed. Your personal information may be disclosed if required by law.

Indirect identifiers such as volunteer's age will be collected to verify their eligibility for the study and telephone number to contact them for availability purposes.

All direct identifiers (name and phone number) will be stored in a separate master sheet with unique codes that will be password-encrypted and stored on password-protected computer, accessible only by the study PI. The data filenames will consist of unique codes (i.e. NIRS000001, NIRS000002, etc.) which will be assigned to each volunteer undergoing data recording/storage to de-identify the data. The data files themselves will not contain identifiable volunteer information stored within so there will be no direct way to link the data to any given volunteer. Recorded data files will be deposited to external hard drives that are not connected to internet by any means. Once study is completed, master key files will be permanently destroyed, as there will be no need for further contact with the volunteers in this study.

The master sheet of volunteer information with unique codes will be password-encrypted and stored on password-protected computer, accessible only by the study PI. Recorded data files will be deposited to external hard drives that are not connected to internet by any means. The data files themselves will not contain identifiable volunteer information

stored within so there will be no direct way to link the data to any given volunteer and they will only be accessible to the PI and research team.

The University of Manitoba Biomedical Research Ethics Board may review records related to the study for quality assurance purposes.

All records will be kept in a locked secure area and only those persons identified will have access to these records. If any of your medical/research records need to be copied to any of the above, your name and all identifying information will be removed. No information revealing any personal information such as your name, address or telephone number will leave HSC.

The data collected from you during this study may be shared in an anonymized or de-identified form with academic journals for publication purposes or other researchers according to international guidelines. The data may also be stored by the academic journal under an open access policy in which case it may be used by other researchers for further data analysis and research purposes.

**Registry Databank**

ClinicalTrials.gov is a website that provides information about federally and privately supported clinical trials. A description of this clinical trial will be available on http://ClinicalTrials.gov. This website will not include information that can identify you. At most, the website will include a summary of the results. You can search this website at any time.

**Voluntary Participation/Withdrawal from the Study**

Your decision to take part in this study is voluntary. You may refuse to participate or you may withdraw from the study at any time. Your decision not to participate or to withdraw from the study will not affect your care at this centre. If the study staff feel that it is in your best interest to withdraw you from the study, they will remove you without your consent.

We will tell you about any new information that may affect your health, welfare, or willingness to stay in this study.

Potential research participants that are students or employees and decide not to participate, their performance evaluation will not be affected by their decision.

**Medical Care for Injury Related to the Study**

In the case of injury or illness resulting from this study, necessary medical treatment will be available at no additional cost to you.

You are not waiving any of your legal rights by signing this consent form nor releasing the investigator(s) or the sponsor(s) from their legal and professional responsibilities.

**Questions**

You are free to ask any questions that you may have about your treatment and your rights as a research participant. If any questions come up during or after the study or if you have a research-related injury, contact the study doctor and the study staff: Dr. Frederick A. Zeiler at (431) 355-7461.

For questions about your rights as a research participant, you may contact The University of Manitoba, Bannatyne Campus Research Ethics Board Office at (204) 789-3389

Do not sign this consent form unless you have had a chance to ask questions and have received satisfactory answers to all of your questions.

**Statement of Consent**

I have read this consent form. I have had the opportunity to discuss this research study with Dr. Frederick A. Zeiler and or his/her study staff. I have had my questions answered by them in language I understand. The risks and benefits have been explained to me. I believe that I have not been unduly influenced by any study team member to participate in the research study by any statements or implied statements. Any relationship (such as employer, supervisor, or family member) I may have with the study team has not affected my decision to participate. I understand that I will be given a copy of this consent form after signing it. I understand that my participation in this study is voluntary and that I may choose to withdraw at any time. I freely agree to participate in this research study.

I understand that information regarding my personal identity will be kept confidential, but that confidentiality is not guaranteed. I authorize the inspection of any of my records that relate to this study by The University of Manitoba Research Ethics Board for quality assurance purposes.

By signing this consent form, I have not waived any of the legal rights that I have as a participant in a research study.

Participant signature: ___________________________ Date: ___________________
(day/month/year)

Participant printed name: ______________________________

I, the undersigned, have fully explained the relevant details of this research study to the participant named above and believe that the participant has understood and has knowingly given their consent

Printed Name: ______________________________________ Date: ___________________
(day/month/year)

Signature: ______________________________

Relationship (if any) to study team members: _______________________
